# Effects of pretreatment during drying on the antioxidant properties and color of selected tomato varieties

**DOI:** 10.1002/fsn3.581

**Published:** 2018-01-12

**Authors:** Rosemary Mwende, Willis Owino, Samuel Imathiu

**Affiliations:** ^1^ Department of Food Science and Technology Jomo Kenyatta University of Agriculture and Technology Nairobi Kenya

**Keywords:** lightness, lycopene, pretreatment, total phenolics, β carotene

## Abstract

Drying is essential in lowering the water activity and increasing the shelf stability of perishables. Thus, this study investigated the effect of pretreatment on the retention of the antioxidant properties and color of four tomato varieties; that is, Anna F1, Kilele, Prostar F1, and Riogrande during drying. Prepared quarters were treated by spraying with 0.5% sodium metabisulfate, 0.5% calcium chloride, and distilled water. The quarters were oven dried at 50°C, 60°C, and 70°C to 13% moisture content. Lycopene, β carotene, total phenolics, color, and moisture content were determined in both the fresh and dried samples. Initial moisture content among the four varieties did not differ significantly and ranged between 94.2 and 94.6%. Results showed that the main effects were significant (*p* < .05) on all measurable variables. Significantly (*p* < .05) higher retention levels in lycopene, β carotene, total phenolics, and lightness was observed in chemically pretreated samples compared to the control during drying.

## 1 INTRODUCTION

Tomato *(Lycopersicon esculentum mill)* is among the most highly consumed and popular vegetable in the world (Hanson et al., 2004). Nutritionally, tomatoes are rich sources of antioxidant compounds such as β carotene, lycopene, ascorbic acid, and phenolic compounds (Georgé et al., 2011). Lycopene and β carotenes are carotenoids responsible for the red, yellow, and orange colors of most plants which cannot be synthesized by animals in vivo and require consumption in the diet (Eldahshan et al., 2013). Scientific evidence shows that consumption of these phytonutrients on a regular basis contribute to significant health benefits such as prevention against diseases such as prostate cancer, age degenerative diseases, and cataracts (Gümüsay, Zoran, Ercal, & Demirkol, 2015). This is attributed to their ability to quench singlet oxygen and trap peroxyl radicals (Gümüsay et al., 2015).

Tomato production in Kenya has increased in the recent past with the adoption of greenhouses. Production increased from 20,985 Ha in 2013–24,074 Ha in 2014, representing a 15% increase in area under production (MOA, 2015). However, being climacteric crops, tomatoes are inherently perishable with a shelf life of 8–12 days in their fresh state after harvest (Ahmed, Islam, Sarker, Hasan, & Mizan, 2016). This has led to extensive postharvest losses in the product that have been estimated to be as high as 50%. These losses translate to a subsequent imbalance in supply and demand and consequential losses in income to both small‐ and large‐scale farmers. In order to sustain surplus harvest, appropriate postharvest preservation methods are needed to extend the commodity's shelf life. One such technology is drying which lowers the moisture content and consequently the water activity of food to a level that does not support bacterial and mold growth (Joshi, Orsat, & Raghavan, 2009). However, during drying some nutrients are degraded by heat thus affecting the quality and acceptance of the final product (Taylor, Goula, Adamopoulos, Goula, & Adamopoulos, 2010). The degree and extend of oxidation as well as isomerization is directly related to the duration and intensity of heating (Eldahshan et al., 2013). As a result, there is need to enhance the rate of drying to ensure maximum retention of antioxidant molecules in tomatoes as well as reduce oxidative, nonenzymatic, and isomerization reactions thus protect these molecules from degradation. Since color, lycopene, and total phenolic content in tomato are regarded as good quality indicators of the dehydration process (Santos‐Sánchez, Valadez‐Blanco, Gómez‐Gómez, Pérez‐Herrera, & Salas‐Coronado, 2012), minimization of quality degradation in these indicators is paramount. In this regard, osmotic assisted dehydration with compounds such as calcium chloride and sodium chloride has been shown to increase the drying rate in plant tissues by enhancing water mobility. It has also been reported that osmotic assisted dehydration improves general product quality (Azoubel & Oliveira, 2008) by preserving the nutritional, sensorial, and functional properties of the food matrix (Kennedy, 2007). However, to ensure water activity below 0.9 is achieved, convective drying methods should be carried out after osmotic dehydration (Kennedy, 2007). Therefore, this study investigated the effects of pretreatment on lycopene, β carotene, total phenolics, and color during oven drying of four tomato varieties.

## 2 MATERIAL AND METHODS

### 2.1 Tomato growing, harvesting, and sample preparation

The four tomato varieties under study were grown in a greenhouse in Juja, Kenya (latitude: 1°10′S, longitude: 37°7′E, altitude: 1416 M) in the Jomo Kenyatta University of Agriculture and Technology (JKUAT) experimental farm from March 2016 to July 2016. The varieties planted were Anna F1 and Prostar F1 (indeterminate varieties) as well as Kilele F1 and Riogrande (determinate varieties). Soil replenishment of nutrients and the control of undesirable pest and diseases followed commercial practices of tomato production. Pruning was carried out to remove undesirable side branches after every 2 weeks. Tomato varieties of uniform maturity (red ripe maturity stage), color, size, and shape were randomly selected for the study. Color selection was based on the USDA color scale (1997) where at least 90% of the surface of the tomato was red.

Nine kilograms of each tomato variety was harvested after reaching red ripe maturity stage, weighed using a digital balance and subdivided into three equal batches of three kilograms each. The harvested tomatoes were washed in running tap water to remove debris and dried using a dry cloth. Each tomato was longitudinally sliced into four equal quarters using a manual slicer. The first batch was further subdivided into three equal batches each one kilogram and treated by spraying with 0.5% w/v sodium metabisulfate (0.5% N.M). The second and third batches were similarly subdivided and sprayed with 0.5% w/v calcium chloride (0.5% C.C) and distilled water (control), respectively. The treated samples were allowed to stand for 20 min to drain away excess spray.

### 2.2 Drying procedure

Drying was carried out in an oven drier (memmert UF 110 model; Germany).

### 2.3 Oven drying

The oven drier was operated for 1.5 hr before drying to achieve steady‐state conditions. The batches were separately placed in a single layer on 2 mm aperture 60 cm × 30 cm removable aluminum meshed trays and dried at 50°C, 60°C, and 70°C. Drying was done to final moisture content of ~13%.

At the end of each drying procedure, moisture content of the samples was determined and the dried samples were put into zip lock bags and stored at −20°C away from light until further analysis. All the experiments were carried out in triplicates and the results expressed on dry weight basis (db) except moisture content which was expressed on fresh weight basis.

### 2.4 Moisture content determination

Moisture content in the fresh and dried tomato samples was analyzed according to the (A.O.A.C, 1999) and the % moisture content calculated based on the formula:

%moisture content=change in weight/sample weight×100

### 2.5 Color determination

The color of both the fresh and dried samples was determined following the method detailed by Dadalı, Kılıç Apar, and Özbek (2007) with some modifications using a hunter lab color difference meter (Minolta, Tokyo, Japan). The instrument was standardized with a black and white ceramic plate before use. The color of the samples was measured at three regions along the blossom end, the stem end, and around the equatorial region. Reflected colors L*, a*, and b* were determined directly as displayed on the color meter screen. L* values were used as an indicator of lightness in the samples analyzed.

### 2.6 Determination of total phenolic compounds

Folin ciocalteu method was used to determine the amount of total phenolic compounds as described by Ainsworth and Gillespie (2007) with slight modifications. Gallic acid was used as the standard. 2 g of each tomato sample was crushed in a pestle and mortar and put in a vial after which 50 ml of cold methanol was added. The sample was vortexed for 3 hr and incubated for 72 hr at 25°C away from light. The extract was filtered to remove the debris and centrifuged at 13,000*g* for 10 min at 25°C and the supernatant collected. A 1 ml of the extract was passed through a 0.45 μl membrane filter. A 2 ml of 10% (V/V) Folin ciocalteu reagent was added and vortexed after which 4 ml of 0.7 mol/L Na_2_CO_3_ solutions was added. Folin ciocalteu was added before Na_2_CO_3_ to prevent air oxidation of the phenols in the extract. The mixture was allowed to stand for 2 hr at 25°C and the absorbance measured at 765 nm using UV‐vis spectrometer (Shimadzu UV Vis 1800, Tokyo, Japan model). A standard curve was plotted from the blank corrected absorbance of the gallic acid standard. The amount of total phenolic content was expressed as gallic acid equivalents GAE) per 100 g of the sample.

### 2.7 β carotene and lycopene content determination

The method suggested by Chen (2005) was employed with some modification for the determination of lycopene and β carotene. About 5 g of crushed tomato sample was weighed using a digital balance and put into amber bottles after which 50 ml of hexane‐acetone‐ethanol solution (2:1:1 v/v/v) containing 1% BHT (w/v) was added to solubilize lycopene. The content was then agitated for 20 min after which 15 ml of distilled water was added to the mixture and mixed for 10 min. The solution was separated into polar and a nonpolar phase using a separating funnel. A 50 ml of the upper hexane layer was collected and 1.5 ml of it was microfiltered using 0.45 μl membrane filters. The extracts were stored at −20^°^C until high‐performance liquid chromatography (HPLC) analysis. β carotene and lycopene were analyzed using a Shimadzu brand HPLC (10A model;Tokyo, Japan) fitted with a LC‐10AS pump, CTO‐10A Column oven, SPD‐10AV UV‐Vis detector and a C18 ODS nonpolar column. The mobile phase contained acetonitrile: methanol: dichloromethane: hexane (40:20:20:20, v/v/v/v) at a flow rate of 1.5 ml/min. Injection volume used was 20 μl, whereas the detection wavelength for lycopene was 470 nm and that of β carotene was 445 nm. The temperature of the oven was maintained at 30^°^C. Lycopene and β carotene standard concentrations were prepared in hexane. Quantification was done using chromatographic peak areas generated to determine the lycopene content and β carotene content. Lycopene and β carotene in the sample was identified by comparing the retention time of pure lycopene and β carotene.

### 2.8 Statistical analysis

The experiment was carried out in triplicate and data subjected to analysis of variance (ANOVA) using Stata SE version 12 (Stata Corp LP, TX, USA). ANCOVA which combines features of both ANOVA and regression was applied to test effects of pretreatments, variety, and temperatures during drying. When the coefficient of the interaction term was significant (*p* < .05), it was concluded that there was a significant difference between treatments. One‐way ANOVA was performed where treatment outcomes needed to be compared. Means were separated using Bonferroni adjustment at 95% level of significance.

## 3 RESULTS AND DISCUSSION

### 3.1 Drying time required to attain stable moisture content

The initial moisture content (m. c) in the four varieties was not significantly different (*p* > .05) and ranged between 94.2 and 94.6% fresh weight basis (f. w). Drying was done to a final m. c of ~ 13% (f. w). The time required to reach ~13% m. c f. w during oven drying is as shown in Table 1. It was observed that both temperature and chemical pretreatment had a significant effect on the drying time compared to the control (*p* < .05). However, drying time required in 0.5% C.C and 0.5% N.M pretreated samples was not significantly different when drying at 50°C in all the varieties under study. Contrastingly, drying time in 0.5% N.M and 0.5% C.C samples during drying at 60°C in Anna F1 and Prostar F1 was significantly different (*p* < .05). At the same temperature (60°C), the drying time in pretreated determinate varieties (Kilele and Riogrande) was not significantly different (*p* > .05). On the other hand, the drying time in the pretreated determinate varieties dried at 70^°^C was significantly different (*p* < .05) whereas the pretreated indeterminate varieties drying time was not significantly different (*p* > .05) at the same drying temperature. Overall, chemically pretreated samples exhibited shorter drying time compared to the control. This can be attributed to the ability of osmotic solutions to cause a higher dehydration force compared to the control hence the shorter drying time (Dalben et al., 2012). This phenomenon was important in saving of energy and in maintaining the quality of the dried product. Notably, in this study, raising drying temperature from 50°C to 70°C reduced drying time at every specific treatment. As a result, the critical moisture content (~ 13% f. w) was attained after a shorter period as shown in Table 1. This was attributed to a greater vapor pressure deficit that resulted after increase in temperature from 50°C to 70°C. Similar findings were observed by Faisal, Tabassum, and Kumar (2013) during hot air drying of potato cubes whereby an increase in moisture migration from the food matrix to the drying medium was observed when drying temperature was raised from 60°C to 80°C. In another study, reduced drying time was observed during drying of collard leaves when drying temperatures were raised from 50°C to 75°C (Alibas, 2009). This was attributed to increased mass transfer associated with increase in temperature.

**Table 1:** Time (min) required to attain 13% m.c (f.w) during oven drying of the four tomato varieties at specific drying temperatures

Tomato variety	Treatment	Oven‐dried samples		
		50°C	60°C	70°C
Anna F1	Control	2886 ± 1^a^	1446 ± 3^a^	1032 ± 7^a^
	0.5% C.C	2826 ± 2^b^	1368 ± 3^b^	972 ± 8^b^
	NM	2838 ± 5^b^	1344 ± 4^c^	948 ± 5^b^
Kilele	Control	2826 ± 4^a^	1326 ± 4^a^	954 ± 1^a^
	0.5 %C.C	2724 ± 3^b^	1236 ± 1^b^	912 ± 7^b^
	0.5 %NM	2718 ± 2^b^	1224 ± 1^b^	882 ± 3^c^
Prostar F1	Control	2844 ± 5^a^	1404 ± 4^a^	996 ± 3^a^
	0.5 %C.C	2784 ± 4^b^	1338 ± 8^b^	930 ± 3^b^
	0.5 %NM	2784 ± 11^b^	1290 ± 9^c^	924 ± 2^b^
Riogrande	Control	2772 ± 8^a^	1230 ± 4^a^	858 ± 8^a^
	0.5 %C.C	2670 ± 3^b^	1176 ± 2^b^	798 ± 5^b^
	0.5 %NM	2664 ± 3^b^	1158 ± 6^b^	762 ± 7^c^

Data are mean values ± *SE* of three replicates. Entries in the same column at a given variety followed by the same superscript letter are not significantly different (*p* > .05). Mean values were separated using Bonferroni adjustment.

### 3.2 Color changes during drying

Color is an important quality indicator in most food products and plays a key role in consumer preference during purchase (Ringeisen, Barrett, & Stroeve, 2014). Thus, a change in the color of a product during processing is generally associated with decrease in the quality and marketability of that product. L* indices were used to characterize the coloration of both fresh and dried tomato samples in this study as shown in Table 2. Fresh tomato varieties under this study had an L* in the range of 41.12–42.33. Significant decrease in lightness relative to the fresh samples occurred in all the samples upon drying as follows: 0.5% N.M < 0.5% C.C< control as shown in Table 2.

**Table 2:** L* values in fresh and oven‐dried tomato varieties dehydrated at 50°C, 60°C, and 70°C

Tomato variety	Treatment	Oven‐dried samples		
		50°C	60°C	70°C
Anna F1	Fresh	42.21 ± 0.4^a^	42.21 ± 0.4^a^	42.21 ± 0.4^a^
	Control	26.13 ± 0.47^b^	27.31 ± 0.35^b^	26.93 ± 0.43^b^
	0.5% C.C	29.71 ± 0.81^c^	30.11 ± 0.26^c^	29.07 ± 0.23^c^
	0.5% N.M	32.34 ± 0.23^d^	33.45 ± 0.26^d^	37.56 ± 0.28^d^
Kilele	Fresh	41.12 ± 0.20^a^	41.12 ± 0.20^a^	41.12 ± 0.20^a^
	Control	26.63 ± 0.26^b^	27.76 ± 0.47^b^	26.48 ± 0.43^b^
	0.5% C.C	28.72 ± 0.41^c^	27.88 ± 0.31^b^	28.46 ± 0.45^c^
	0.5% N.M	34.01 ± 0.37^d^	33.22 ± 0.34^c^	37.74 ± 0.25^d^
Prostar F1	Fresh	42.33 ± 0.29^a^	42.33 ± 0.29^a^	42.33 ± 0.29^a^
	Control	26.58 ± 0.60^b^	28.58 ± 0.29^b^	27.44 ± 0.35^b^
	0.5% C.C	29.74 ± 0.16^c^	27.2 ± 0.23^b^	28.42 ± 0.25^b^
	0.5% N.M	33.15 ± 0.40^d^	32.46 ± 0.56^c^	35.45 ± 0.26^c^
Riogrande	Fresh	41.61 ± 0.34^a^	41.61 ± 0.34^a^	41.61 ± 0.34^a^
	Control	26.78 ± 0.78^b^	26.68 ± 0.76^b^	31.4 ± 0.28^b^
	0.5% C.C	29.11 ± 0.28^c^	29.05 ± 0.31^c^	32.24 ± 0.56^bc^
	0.5% N.M	30.92 ± 0.22^d^	36.84 ± 0.54^d^	34.02 ± 0.64^c^

Data are mean values ± SE of three replicates entries in the same column in a given variety followed by the same superscript letter is not significantly different (*p* > .05).Mean values were separated using Bonferroni adjustment.

Generally, it was observed that the lowest degree of darkening was found in samples pretreated with 0.5% N.M and the highest degree of darkening was in the control samples after oven drying. This indicated that chemical pretreatment was preventive against oxidation that is characterized by formation of darkened products. It has been reported that sulfites reduce o‐quinones to colorless di‐phenols thus prevent browning (Sgroppo, Vergara, & Tenev, 2010). The low luminosity in the control samples was indicative of possible darkening that is characteristic of enzymatic and nonenzymatic reactions that occur during heat processing Luterotti, Bicanic, and Markovi(2014) therefore, indicating that the effect of 0.5% N.M in mitigating darkening was greater compared to that of the control and 0.5% C.C. It has been reported that decrease in L* value during drying may be associated with carotenoid degradation, maillard, and nonenzymatic reactions (Nisha, Singhal, & Pandit, 2011). Similar findings were observed during tomato puree processing where L* value significantly decreased during heat processing (Nisha et al., 2011).

### 3.3 Effect of pretreatment on the total phenolic content of tomato varieties during oven drying

**Figure 1:**
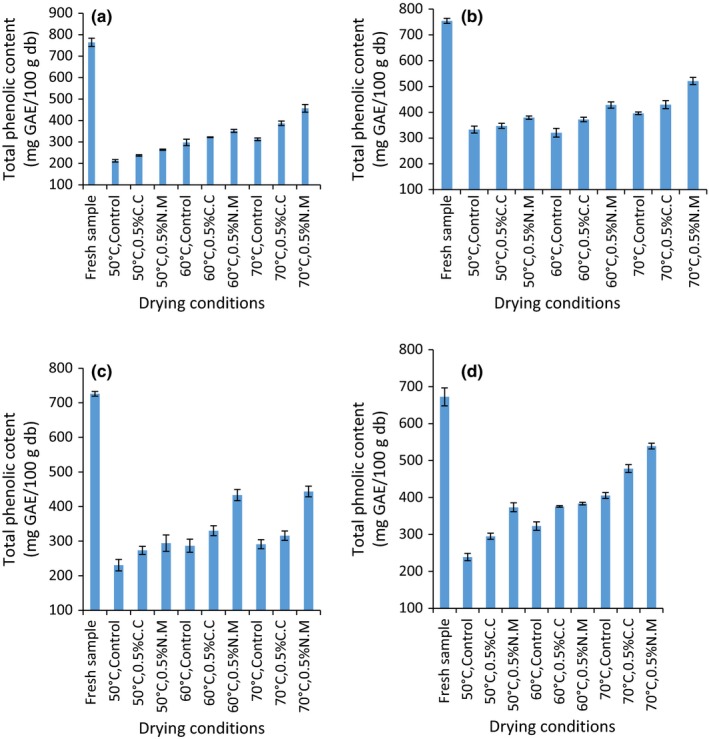
Total phenolic content of fresh and pretreated tomato samples oven dried at 50°C, 60°C, and 70°C in (a) Anna F1 (b) Kilele (c) Prostar F1 (d) Riogrande varieties expressed in mg/100 g DW. Plotted data are mean ± standard error of three replicates

The effect of pretreatment on the total phenolic content (TPC) content in oven‐dried tomato samples is shown in Figure 1. The initial TPC in the four fresh tomato under study was significantly different (*p* = .0174) and occurred in the range of 672 ± 24.30–764.28 ± 18.94 mg GAE/100 g DW. Riogrande tomato variety had the least TPC (672 mg GAE/100 g DW), whereas Anna F1 had the highest (764 g GAE/100 g DW) TPC content. The differences in the phenolic content among the four varieties under this study may be attributed to varietal differences that have been identified as influencing factors in the synthesis of phenolic compounds in plants (Hanson et al., 2004). The higher phenolic content in Anna F1 variety as compared to that of Riogrande variety may also be attributed to its thick skin representing a higher skin to flesh ratio as compared to Riogrande variety which has a thinner skin. Studies show that most phenolic compounds are concentrated on the skin surface of a fruit (Dadalı et al., 2007). Thus, thick skinned tomato varieties have a higher TPC content than their thin skinned counterparts.

Statistical analysis showed that there was a significant difference (*p* < .05) between the fresh samples and the oven‐dried samples. The interaction effect between variety, drying temperature and chemical pretreatment on the TPC content in oven‐dried samples was found to be highly significant (*p* = .0006; *F* = 3.40; *df* = 12). As a main effect, chemical pretreatment was found significant for TPC (*p* = .0001; *df* = 2; *F* = 218.11). In this regard, the TPC retained in the dried samples after chemical pretreatment occurred in the range of 212–405, 237–478, and 263–539 mg GAE/100 g DW in the control, 0.5% C.C, and 0.5% N.M, respectively. This corresponds to maximum percentage retention of 60%, 71%, and 80% for the corresponding chemical pretreatments relative to the fresh. The higher minimization of phenolic degradation through pretreatment as compared to the control may be attributed to the ability of sodium metabisulfate and calcium chloride to retard oxidative reactions and tissue damage (Sgroppo et al., 2010) that may cause irreversible changes in the quality of dried produce. On the other hand, the percentage retention was 27%–59%, 42%–69%, 31%–61%, and 35%–80% for Anna F1, Kilele, Prostar F1, and Riogrande variety, respectively, depending on the drying temperatures and chemical pretreatment applied. This showed that phenolic compounds were best preserved in Riogrande variety and least in Anna F1. This may be associated with lower drying time experienced during drying in Riogrande variety as compared to Anna F1 (Table 1). Noteworthy, the effect of temperature on the final TPC content was highly significant (*p* < .05; *df* = 2; *F* = 325.93). Drying at higher temperatures corresponded to overall higher retention of TPC as compared to drying at lower temperatures. In this aspect, TPC retention occurred in the order of 70°C > 60°C > 50°C in all the varieties under this study. This represented percentage TPC retention of 27%–55%, 38%–59%, and 40%–80% when drying at 50°C, 60°C, and 70°C, respectively. In all the varieties studied, the best drying conditions for maximum phenolic retention occurred after drying at 70°C accompanied with 0.5% N.M pretreatment. This might be due to shorter drying time at higher temperature thus reduced exposure to oxygen and heat. Also, it is possible that at higher temperatures, cellular integrity of the sample was compromised thus facilitating higher extractability of TPC as compared to lower drying temperatures. Dadalı et al.(2007) suggested that high drying temperatures (80°C) releases phenolic compounds bound to other cell components in most vegetables during thermal processing.

### 3.4 Effect of pretreatment on the β carotene content of tomato during oven drying

**Figure 2:**
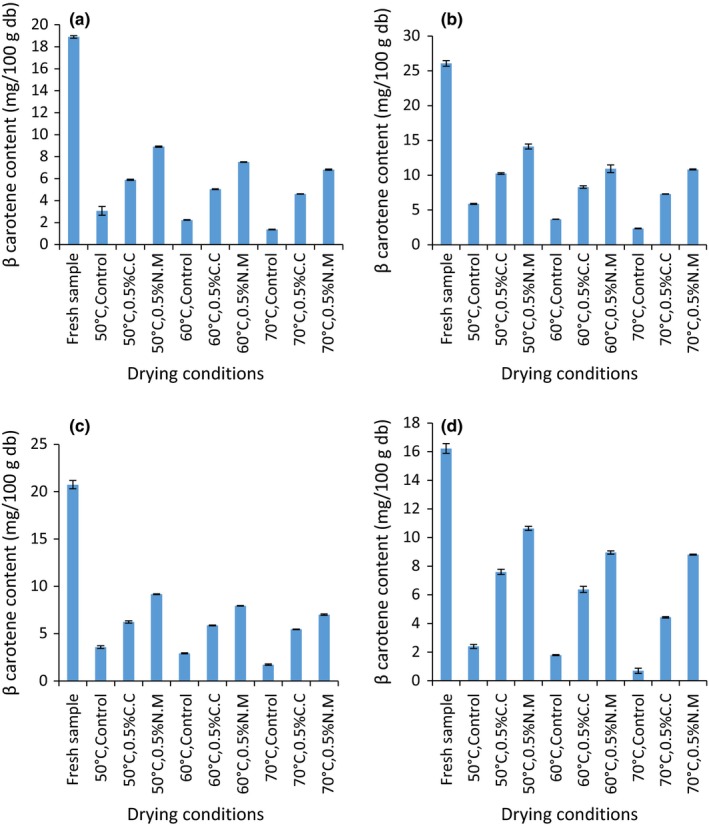
β carotene content expressed in mg/100 g in pretreated (a) Anna F1, (b) Kilele, (c) Prostar F1, and (d) Riogrande tomato varieties oven dried at 50°C, 60°C, and 70°C. Plotted data are mean standard error of three replicates

Figure 2 shows the effect of pretreatment on the β carotene content in tomatoes during oven drying. The β carotene content in the fresh tomato varieties was found to be significantly different (*p* = .0001). β carotene content in fresh tomato varieties under study was 18.90 ± 0.11, 26.07 ± 0.39, 20.74 ± 0.43, and 16.22 ± 0.34 mg/100 g DW corresponding to about 0.97, 1.35, 1.07, and 0.84 mg/100 g fresh weight (fw) for Anna F1, Kilele, Prostar F1, and Riogrande, respectively. Similar findings were reported by Georgé et al. (2011) who found β carotene content to occur in the range of 0.6 ± 0.1–1.0 ± 0.1 mg/100 g f. w in tomatoes.

Statistically, there was an interaction effect between drying temperatures, pretreatment, and the variety on the content of β carotene in the dried samples (*df* = 35; *F* = 427.34; *p* = .0001). The effect of the main effects including temperature (*p* = .0001) and pretreatment (*p* = .0001) were found to be significant on the β carotene content retained after oven drying. Higher β carotene retention values were observed in the pretreated‐dried samples as compared to the control. The percentage retention values were 4.30–22.56, 24.41–46.85, and 33.81–65.58 in the control, 0.5% C.C, and 0.5% N.M, respectively. On the other hand, 7.28–47.16, 8.97–54.20, 8.33–44.21, and 4.30–65.58% of β carotene relative to the fresh was retained in Anna F1, Prostar F1, and Riogrande varieties, respectively. Based on temperature, 16.26–65.58, 11.05–55.21, and 4.30–41.52% of the initial β carotene was retained after drying at 50, 60, and 70°C in all the varieties studies. A key observation, however, was that raising the drying temperature during oven drying from 50°C to 70°C resulted in a higher β carotene loss despite shorter exposure to drying air required to attain stable moisture content. This shows that β carotene retention was highest in samples dried at 50°C as compared to those dried at 60°C and 70°C despite increased drying time. This was attributed to possible higher isomerization and oxidation rate at 70°C as compared to drying at 50°C (Eldahshan & Singab, 2013). This therefore suggests that for maximum β carotene retention the recommended drying conditions would 50°C accompanied with 0.5% sodium metabisulfate pretreatment in all the varieties under this study.

### 3.5 Effect of pretreatment on the lycopene content of tomato during oven drying

**Figure 3:**
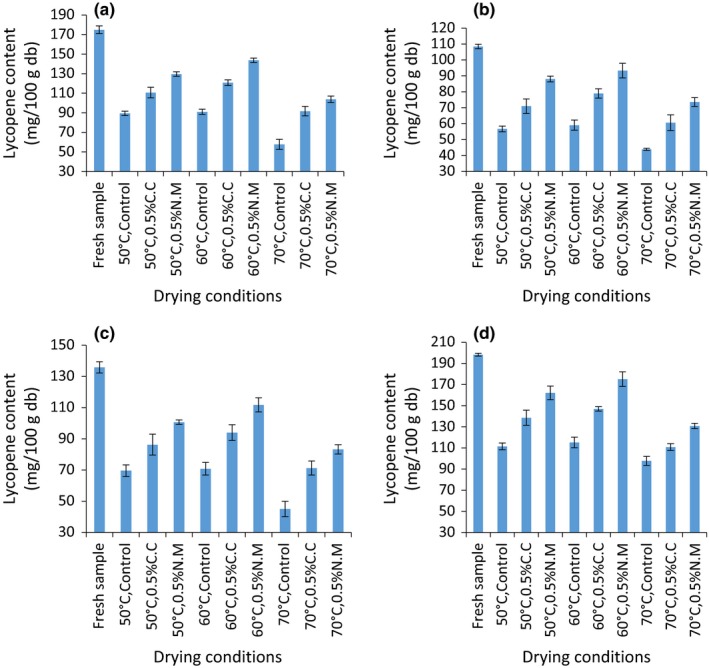
Lycopene content of fresh and pretreated tomato samples oven dried at 50°C, 60°C, and 70°C in (a) Anna F1, (b) Kilele, (c) Prostar F1, and (d) Riogrande varieties expressed in mg/100 g DW. Plotted data are mean ± standard error of three replicates

The effect of pretreatment on the lycopene content in tomatoes during oven drying is shown in Figure 3. The lycopene content among the fresh tomato varieties studied differed significantly (*p* = .001). The lycopene content was 174.86 ± 3.84, 108.46 ± 1.36, 135.80 ± 3.60, and 198.25 ± 1.39 mh/100 g DW for Anna F1.Kilele, Prostar F1, and Riogrande, respectively. Varietal differences were linked to the differences in the lycopene content since the four varieties were grown under the same conditions (Tigist & Workneh, 2013). This was comparable to findings of Olufemi, Pamela, Ibitoye, and Olubunmi (2009) who analyzed eight cultivars of tomato and found lycopene to occur in the range of 70.25–147.29 μg/gf. w which corresponds to about 117–245.33 mg/100 g db.

ANCOVA analysis showed no interaction effect between temperature, variety, and drying temperature on the lycopene content in the dried samples (*p* = .5375;*F* = 0.91; *df *= 12). However, the main effects including temperature (*df* = 2; *F* = 144.16, *p* = .0001), chemical pretreatment (*df* = 2; *F* = 294.05; *p* = .0001), and variety (*df* = 3; *F* = 405.08; *p* = .0001) were found to have a significant effect on the lycopene content in the dried samples. It was observed that pretreatment significantly enhanced retention of lycopene in all the oven‐dried samples as compared to the control. The % retention of lycopene was 33.05–58.11, 52.44–74.13, and 59.38–88.33% in the control, 0.5% C.C, and 0.5% N.M pretreated samples, respectively. Sodium metabisulfate has been reported to retard and inhibit oxidation that is mainly linked to adverse degradation of carotenoids (Sahin et al., 2011). This is attributed to their ability to act as reducing agents during oxidative reactions (Sra & Sandhu, 2011). With regard to temperature, lycopene content was found to be best retained after drying at 60 °C whereby the % retention varied between 52.01 and 88.33%. The least % retention occurred after drying at 70°C whereby the retention was 33.05–67.83%, whereas intermediate retention (51.16–74.13%) occurred in samples dried at 50°C across all the four varieties studied. Similar findings were reported by Capanoglu, Beekwilder, Boyacioglu, De Vos, and Hall (2010) who quantified lycopene retention after heat processing of tomatoes into paste to be 88–72% of the initial concentration. Elsewhere, 67% lycopene content was retained after heat processing of Rosso tondo tomato cultivar at 70°C (Lenucci et al., 2010). Therefore maximum lycopene retention occurred in samples dried at 60°C accompanied with 0.5% N. M pretreatment. On the other hand, the lycopene content retained after oven drying in the four varieties studied followed the order Riogrande > Kilele > Prostar F1 > Anna F1. This corresponded with 33.05–82.17, 40.94–85.98, 33.17–82.26, and 49.31–88.33% retention relative to the fresh in Anna F1, Kilele, Prostar F1, and Riogrande, respectively. This phenomenon was largely influenced by the shorter drying time required in Riogrande to achieve stable moisture content as compared to the other varieties under study (Table 1). Longer drying time results in higher exposure of the sample to oxygen and heat. This catalyzes the rate of isomerization and oxidation which result in higher carotenoid loss (Khoo, Prasad, Kong, Jiang, & Ismail, 2011).

## 4 CONCLUSION

Attempt to lower moisture content in Anna F1, Kilele, Prostar F1, and Riogrande tomato varieties to ensure shelf stability resulted in overall reduction in lycopene, β carotene, total phenolic compounds, and color compared to the fresh samples. However, pretreatment with 0.5% N.M and 0.5% C.C significantly preserved the overall quality of dried tomato samples during oven drying as compared to the control. The overall quality of the dried tomato was highly influenced by drying temperature, tomato variety, and chemical pretreatment. Lycopene, total phenolic compounds and β carotene was best retained by drying at 60^°^C (0.5% N.M), 70^°^C (0.5% N.M), and 50°C (0.5% N.M), respectively, in all varieties under study. The degree of darkening was least in the dried samples as follows: 0.5% N.M < 0.5% C.C < control. The study therefore showed that pretreatment is one of the techniques that can be used in controlling undesirable quality changes that occur during tomato drying.

## CONFLICT OF INTEREST

None declared.

## References

[fsn3581-bib-0001] Ahmed, M. F. , Islam, M. Z. , Sarker, M. S. , Hasan, S. K. , & Mizan, R. (2016). Development and performance study of controlled atmosphere for fresh tomato. World Journal of Engineering and Technology, 4(02), 168–175. https://doi.org/10.4236/wjet.2016.42015

[fsn3581-bib-0002] Ainsworth, E. A. , & Gillespie, K. M. (2007). Estimation of total phenolic content and other oxidation substrates in plant tissues using Folin – Ciocalteu reagent. Nature protocols, 2(179337), 875–877. https://doi.org/10.1038/nprot.2007.102 https://doi.org/10.1038/nprot.2007.102 17446889 10.1038/nprot.2007.102

[fsn3581-bib-0003] Alibas, I. (2009). Microwave, vacuum, and air drying characteristics of collard leaves. Drying Technology, 27(11), 1266–1273. https://doi.org/10.1080/07373930903267773

[fsn3581-bib-0004] A.O.A.C . (1999). Official Methods of Analysis. Association of Official Analytical Chemist (pp. 155–159). Arlington, VA., USA: Association of Analytical Communities.

[fsn3581-bib-0005] Azoubel, M. , & Oliveira, F. (2008). Original article Optimisation of osmotic dehydration of “Tommy Atkins “mango fruit. International Journal of Food Science and Technology, 43, 1276–1280. https://doi.org/10.1111/j.1365-2621.2007.01605

[fsn3581-bib-0008] Capanoglu, E. , Beekwilder, J. , Boyacioglu, D. , De Vos, R. C. H. , & Hall, R. D. (2010). The effect of industrial food processing on potentially health‐beneficial tomato antioxidants. Critical Reviews in Food Science and Nutrition, 50(10), 919–930. https://doi.org/10.1080/10408390903001503 21108072 10.1080/10408390903001503

[fsn3581-bib-0009] Chen, C. H. L. B. H. (2005). Stability of carotenoids in tomato juice during processing. European Food Research Technology, 221, 274–280. https://doi.org/10.1007/s00217-005-1155-y

[fsn3581-bib-0010] Dadalı, G. , Kılıç Apar, D. , & Özbek, B. (2007). Color change kinetics of okra undergoing microwave drying. Drying Technology, 25(5), 925–936. https://doi.org/10.1080/07373930701372296

[fsn3581-bib-0501] Dalben, C. , Campos, M. , Carla, A. , Sato, K. , & Tonon, R. V. (2012). Effect of process variables on the osmotic dehydration of star‐fruit slices. Ciência e Tecnologia de Alimentos Campinas, 32(2), 357–365.

[fsn3581-bib-0011] Eldahshan, O. A. , & Singab, A. N. B. (2013). Carotenoids. Journal of Pharmacognosy and Phytochemistry, 2(1), 225–234.

[fsn3581-bib-0012] Faisal, S. , Tabassum, R. , & Kumar, V. (2013). Performance evaluation and process optimization of potato drying using hot air oven. Journal of Food Processing and Technology, 4(10), https://doi.org/10.4172/2157-7110.1000273

[fsn3581-bib-0013] Georgé, S. , Tourniaire, F. , Gautier, H. , Goupy, P. , Rock, E. , & Caris‐veyrat, C. (2011). Changes in the contents of carotenoids, phenolic compounds and vitamin C during technical processing and lyophilisation of red and yellow tomatoes. Food Chemistry, 124, 1603–1611. https://doi.org/10.1016/j.foodchem.2010.08.024

[fsn3581-bib-0014] Gümüsay, O. A. , Zoran, I. S. , Ercal, N. , & Demirkol, O. (2015). Drying effects on the antioxidant properties of tomatoes and ginger. Food Chemistry, 173, 156–162. https://doi.org/10.1016/j.foodchem.2014.09.162 25466007 10.1016/j.foodchem.2014.09.162

[fsn3581-bib-0015] Hanson, P. M. , Yang, R. , Wu, J. , Chen, J. , Ledesma, D. , Tsou, S. C. S. , & Lee, T. (2004). Variation for antioxidant activity and antioxidants in tomato. Journal of the American Society for Horticultural Science, 129(5), 704–711. https://doi.org/10.1079/PGR200444

[fsn3581-bib-0016] Joshi, N. , Orsat, V. , & Raghavan, G. S. V. (2009). Physical attributes of different cuts of tomatoes during hot air drying. Fresh Produce, 3(1), 32–36.

[fsn3581-bib-0017] Kennedy, J. F. (2007). Optimisation of osmotic dehydration of carrot cubes in sucrose‐salt solutions using response surface methodology. European Food Research and Technology, 157–165, https://doi.org/10.1007/s00217-006-0395-9

[fsn3581-bib-0018] Khoo, H. , Prasad, K. N. , Kong, K. , Jiang, Y. , & Ismail, A. (2011). Carotenoids and their isomers: Color pigments in fruits and vegetables. Molecules, 16, 1710–1738. https://doi.org/10.3390/molecules16021710 21336241 10.3390/molecules16021710 PMC6259627

[fsn3581-bib-0019] Lenucci, M. S. , Caccioppola, A. , Durante, M. , Serrone, L. , Leonardo, R. , & Dalessandro, G. (2010). Optimisation of biological and physical parameters for lycopene supercritical CO_2_ extraction from ordinary and high‐pigment tomato cultivars. Journal of the Science of Food and Agiculture, 90, 1709–1718. https://doi.org/10.1002/jsfa.4006 10.1002/jsfa.4006 20564441

[fsn3581-bib-0020] Luterotti, S. , Bicanic, D. , & Markovi, K. (2014). Carotenes in processed tomato after thermal treatment. Food Control, 40, 4–11. https://doi.org/10.1016/j.foodcont.2014.06.004

[fsn3581-bib-0021] MOALF (2015). Ministry of Agriculture Livestock & Fisheries (MOALF), Republic of Kenya. 2015 Annual Report

[fsn3581-bib-0022] Nisha, P. , Singhal, R. S. , & Pandit, A. B. (2011). Kinetic modelling of colour degradation in tomato puree (*Lycopersicon esculentum* L.). Food Bioprocess Technology, 4, 781–787. https://doi.org/10.1007/s11947-009-0300-1

[fsn3581-bib-0023] Olufemi, A. , Pamela, T. A. , Ibitoye, E. , & Olubunmi, D. (2009). Lycopene content in tomatoes (*Lycopersicon esculentum mill*): Effect of thermal heat and its health benefits. Fresh Produce, 3(1), 40–43.

[fsn3581-bib-0024] Ringeisen, B. , Barrett, D. M. , & Stroeve, P. (2014). Energy for sustainable development concentrated solar drying of tomatoes. Energy for Sustainable Development, 19, 47–55. https://doi.org/10.1016/j.esd.2013.11.006

[fsn3581-bib-0025] Sahin, F. H. , Aktas, T. , Orak, H. , Ulger, P. , Sahin, H. , Aktas, T. , … Ulger P. (2011). Influence of pretreatments and different drying methods on color parameters and lycopene content of dried tomato. Bulgarian Journal of Agricultural Science, 17(6), 867–881.

[fsn3581-bib-0026] Santos‐Sánchez, N. F. , Valadez‐Blanco, R. , Gómez‐Gómez, M. S. , Pérez‐Herrera, A. , & Salas‐Coronado, R. (2012). Effect of rotating tray drying on antioxidant components, color and rehydration ratio of tomato saladette slices. LWT ‐ Food Science and Technology, 46(1), 298–304. https://doi.org/10.1016/j.lwt.2011.09.015

[fsn3581-bib-0027] Sgroppo, S. C. , Vergara, L. E. , & Tenev, M. D. (2010). Effects of sodium metabisulphite and citric acid on the shelf life of fresh cut sweet potatoes. Spanish Journal of Agricultural Research, 8(3), 686–693. https://doi.org/10.5424/sjar/2010083-1266

[fsn3581-bib-0028] Sra, S. K. , & Sandhu, K. S. (2011). Effect of processing parameters on physico‐chemical and culinary quality of dried carrot slices. Journal of Food Science and Technology, 48(2), 159–166. https://doi.org/10.1007/s13197-010-0170-6 23572730 10.1007/s13197-010-0170-6 PMC3551070

[fsn3581-bib-0029] Taylor, P. , Goula, A. M. , Adamopoulos, K. G. , Goula, A. M. , & Adamopoulos, K. G. (2010). Kinetic models of β‐carotene degradation during air drying of carrots. Drying Technology, 28(6), 752–761. https://doi.org/10.1080/07373937.2010.482690

[fsn3581-bib-0030] Tigist, M. , & Workneh, T. S. (2013). Effects of variety on the quality of tomato stored under ambient conditions. Journal of Food Science and Technology, 50(3), 477–486. https://doi.org/10.1007/s13197-011-0378-0 24425942 10.1007/s13197-011-0378-0 PMC3602550

